# ^18^F‐FDG uptake in accessory respiratory muscles shows the respiratory effort of patients with pleuroparenchymal fibroelastosis

**DOI:** 10.1002/rcr2.900

**Published:** 2022-01-20

**Authors:** Hiroko Okabayashi, Hiroko Machida, Aiko Masunaga, Hidenori Ichiyasu, Takuro Sakagami

**Affiliations:** ^1^ Department of Respiratory Medicine, Faculty of Life Sciences Kumamoto University Hospital, Kumamoto University Kumamoto Japan

**Keywords:** ^18^F‐fluorodeoxyglucose–position emission tomography/computed tomography, accessory respiratory muscles, pleuroparenchymal fibroelastosis

## Abstract

Patients with pleuroparenchymal fibroelastosis (PPFE) have severe breathlessness even with mild hypoxaemia. In patients with PPFE, accessory respiratory muscles, such as the sternocleidomastoid muscles, are used to maintain ventilation. An intense ^18^F‐fluorodeoxyglucose uptake in accessory respiratory muscles using positron emission tomography/computed tomography reflects the strong respiratory effort of patients with PPFE.

## Graphical abstract

In patients with severe respiratory disease, accessory respiratory muscles, such as the sternocleidomastoid muscles, are used to maintain ventilation. We present a patient with pleuroparenchymal fibroelastosis with intense ^18^F‐fluorodeoxyglucose uptake in accessory respiratory muscles using positron emission tomography/computed tomography reflecting the strong respiratory effort.

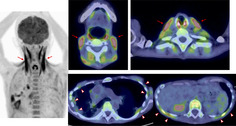

## CLINICAL IMAGE

**FIGURE 1:**
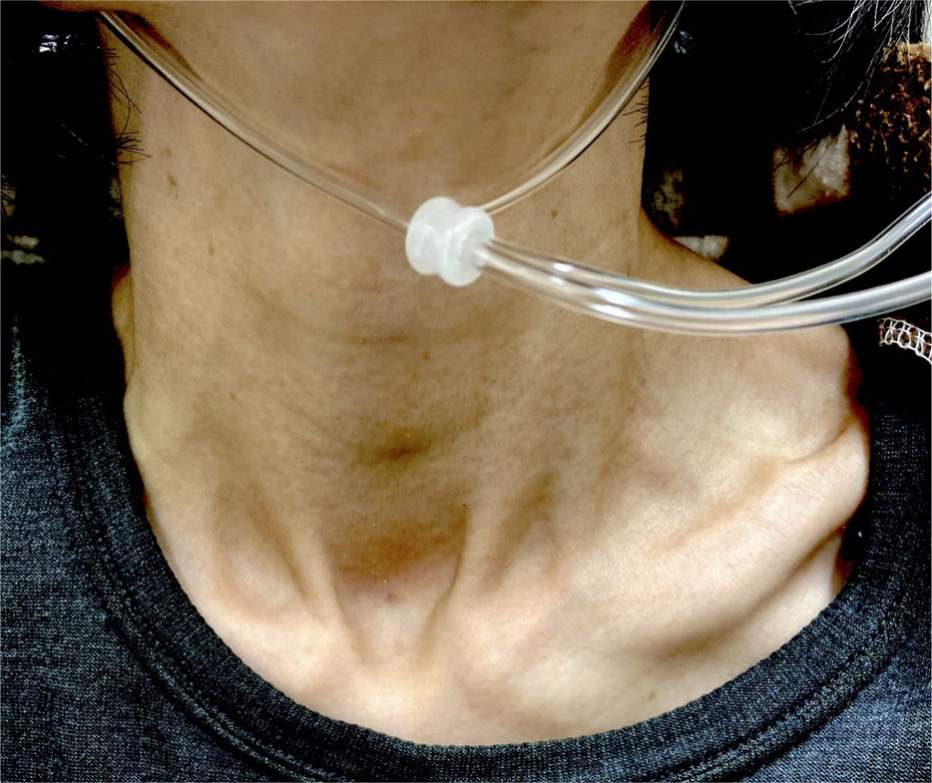
The sternocleidomastoid muscles of the patient were hypertrophic

**FIGURE 2:**
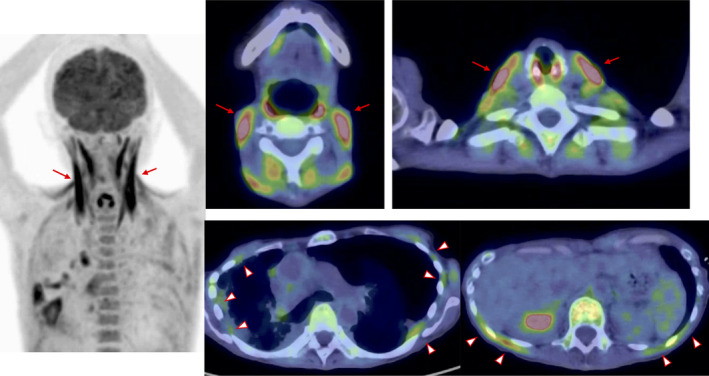
^18^F‐fluorodeoxyglucose (^18^F‐FDG) accumulated in cervical muscles and intercostal muscles (arrowheads). In particular, the uptake of ^18^F‐FDG in sternocleidomastoid muscles (arrows) was high, and the maximum standardized uptake value was 3.2–4.4

A 54‐year‐old female with idiopathic pleuroparenchymal fibroelastosis, gradually progressive over 2 years, was transferred to our hospital for the management of bilateral pneumothoraces complication. She had mild hypoxaemia (pH 7.411, partial pressure of oxygen [PaO_2_] 68.6 mmHg, partial pressure of carbon dioxide [PaCO_2_] 43.3 mmHg; room air), tachypnoea and high respiratory effort on exertion. Her rib cage was flat, showing hypertrophy of the sternocleidomastoid muscles (Figure 1). She also had severe restrictive ventilatory dysfunction (percentage of predicted forced vital capacity 20.1%). Therefore, malignancies were screened for using ^18^F‐fluorodeoxyglucose (FDG)–positron emission tomography/computed tomography, before registration for lung transplantation. The images demonstrated an intense ^18^F‐FDG uptake in the cervical and intercostal muscles, especially in the sternocleidomastoid muscles (Figure 2). In healthy subjects, breathing at rest is usually performed by the diaphragm and external intercostal muscles. However, in patients with severe respiratory disease, accessory respiratory muscles, such as the sternocleidomastoid muscles, are used to maintain ventilation. In studies on patients with chronic obstructive pulmonary disease, ^18^F‐FDG uptake in their respiratory muscles was reported and the uptake correlated with the degree of obstructive ventilatory impairment.^1^, ^2^ This case suggests that a similar result may occur in patients with restrictive ventilatory disease.

## CONFLICT OF INTEREST

None declared.

## AUTHOR CONTRIBUTION

Hiroko Okabayashi was responsible for conceptualization and drafting the manuscript. Hiroko Machida was involved in the acquisition of clinical and radiological data, and drafting the manuscript. Aiko Masunaga, Hidenori Ichiyasu and Takuro Sakagami revised the manuscript. All authors approved the final manuscript.

## ETHICS STATEMENT

The authors declare that an appropriate written informed consent was obtained for the publication of this manuscript and accompanying images.

## Data Availability

Data sharing not applicable to this article as no datasets were generated or analysed during the current study.
